# Prolonged Delivery of Ciprofloxacin and Diclofenac Sodium from a Polymeric Fibre Device for the Treatment of Peridontal Disease

**DOI:** 10.1155/2013/460936

**Published:** 2013-11-14

**Authors:** Deanne Johnston, Yahya E. Choonara, Pradeep Kumar, Lisa C. du Toit, Sandy van Vuuren, Viness Pillay

**Affiliations:** Department of Pharmacy and Pharmacology, Faculty of Health Sciences, University of the Witwatersrand, 7 York Road, Parktown 2193, Johannesburg, South Africa

## Abstract

*In vitro* analysis of drug release and antimicrobial activity of the coblended crosslinked polymeric fibre device (PFD) were investigated. The fibre loaded with ciprofloxacin and diclofenac sodium was comprised of alginate and glycerol crosslinked with barium cations. The pH dependent drug release was evident with ciprofloxacin and diclofenac sodium diffusing from the fibre at pH 4.0 compared to pH 6.8, where the fibre swelled and eroded resulting in zero-order drug release. Agar diffusion studies followed by minimum inhibitory assays were conducted to determine the antimicrobial activity of the device against *Escherichia coli*, *Enterococcus faecalis*, and *Streptococcus mutans*. The antimicrobial activity of the PFD was confirmed in both test assays against all test pathogens. The MIC ranges at pH 4.0 for *E. coli*, *E. faecalis*, and *S. mutans* were 0.5–0.8, 0.4–1.1, and 0.7–2.1 *μ*g/mL, respectively. At pH 6.8, similar efficacies (0.3–0.5 *μ*g/mL for *E. coli* and *E. faecalis* and 0.6–1.0 *μ*g/mL for *S. mutans*) were observed. The effect of varying the plasticizer and crosslinking ion concentration on drug release profile of the fibers was further elucidated and conceptualized using molecular mechanics energy relationships (MMER) and by exploring the spatial disposition of geometrically minimized molecular conformations.

## 1. Introduction

Periodontal disease (PD) describes a chronic bacterial infection affecting the gums and bone supporting the teeth. Such infections often produce toxins leading to a cascade of inflammatory events that if left untreated may lead to permanent tooth loss. The goal when treating PD is the eradication of microorganisms followed by the regeneration of structures destroyed by the disease [1]. Scaling and root planing (SRP) form the crux of periodontal therapy involving the removal of calculus and plaque [2, 3]. SRP combined with home care can lead to positive outcomes, but this may be hard to achieve [4]. Multiple clinical trials have proved that SRP leads to a reduction in microbial load, reductions in bleeding time upon probing, and gain in attachment levels. However, this time-consuming process inevitably leaves behind micro-organisms leading to recolonization [3]. Pharmacological therapy is often used in combination with SRP, delivering one or more chemotherapeutic agents. These may be delivered either systemically or locally within the periodontal pocket. Sustained drug delivery devices can be further subdivided into degradable and nondegradable devices. The device generally consists of a matrix within which the drug is evenly distributed. In nondegradable devices, the drug diffuses from an insoluble nondegradable polymer which needs to be removed after treatment is completed, while degradable devices release the drug via diffusion and matrix erosion and therefore do not need to be removed from the periodontal pocket [5]. Higher levels of drug in the gingival fluid may lead to improved clinical parameters evident with intrapocket delivery systems for even distribution of drug throughout the periodontal pocket [6, 7]. Site-specific drug delivery selectively targets the diseased location with superior treatment results [1]. Furthermore, degradable devices have the added advantage of improved patient compliance as there is no need to remove the device from the pocket [5]. The interaction between bacteria and the immune-inflammatory response may further lead to periodontal tissue destruction which forms the rationale for the treatment with anti-inflammatory and/or antimicrobial agents [8]. The first local drug delivery device for the treatment of periodontal disease was developed by Goodson and coworkers, where a hollow cellulose acetate fibre was filled with tetracycline, releasing 95% of the entrapped drug within 2 hours. Rapid release of the drug from a hollow fibre limited its clinical application [9].

Goodson and co-workers formulated a monolithic fibre where biocompatible polymers, polyethylene, polypropylene, PCL, polyurethane, and cellulose acetate propionate, all released the drug *in vitro *within a day [10]. However, ethylene vinyl acetate provided controlled delivery for up to nine days. The ethylene vinyl acetate fibre demonstrated promising results *in vivo* maintaining constant tetracycline levels (1590 *μ*g/mL) over 10 days. The commercialisation of this tetracycline-loaded ethylene vinyl acetate fibre resulted in the product known as Actisite. Although an improved clinical response is seen in these fibres administered in conjunction with SRP compared to SRP alone [11, 12], problems with the device have been identified. Fibre placement within the pocket is time consuming and requires trained personnel. Ethylene vinyl acetate fibres, a nondegradable device, need to be removed after treatment necessitating a further visit to the therapist. Furthermore, the fibres need to be secured within the pocket using cyanoacrylate glue. These problems limit the use and acceptability of tetracycline-loaded ethylene vinyl acetate fibres [6, 7, 13]. 

Biodegradable fibre devices developed are gravity and electrospun which form a graft matrix-like structure which is placed within the periodontal pocket. Thin fibres are formed resulting in an increased surface area for the diffusion of drug. PCL was the polymer incorporated in fibre formulations releasing gentamicin *in vitro* over 50 days from a nonhomogenised suspension of gravity spun fibres [14] and metronidazole over 19 days from electrospun nanofibres [15]. These novel approaches to fibre production produce a device which can more adequately be compared to a film than a fibre.

This study aimed to formulate a polymeric fibre device (PFD) comprised of alginate, glycerol, and barium chloride with ciprofloxacin and diclofenac sodium incorporated as the antimicrobial and anti-inflammatory model, respectively. Alginate, which has been used extensively in wound care management, was crosslinked with a suitable cation forming the polymeric backbone of the fibre. Alginate, a well known biocompatible polymer used extensively in the food and pharmaceutical industry, is a natural polysaccharide isolated from brown seaweed. The (1,4) linked *α*-L-guluronate and *β*-D mannuronate will be referred to as the G and M monomers. These monomers are joined together to form blocks and may arrange themselves in one of the following patterns (GM), (GG), and (MM). Arrangements of these monomers within these blocks determine the mechanical stability with increased G monomers resulting in high mechanical strength while higher M monomers lead to increased flexibility [16]. 

Multivalent cations bind to the carboxylic groups on the G monomers on two adjacent chains crosslinking the solution into a gel network [17]. Haug and Smidsrød experimentally determined that the concentration of cations that needed to induce gelation and precipitation of sodium alginate prepared from the *Laminaria digitata* and *Laminaria hyperborean* species in increasing order [18, 19] was:

(1) Ba>Pb>Cu>Sr>Cd>Ca>Zn>Ni>Co>Mn,Fe>Mg.    

For the proposed fibre formulation, barium chloride was selected as a suitable cation. Bajpai and Sharma determined that barium chloride is more stable in a medium of varying pH compared to calcium chloride, and the large barium ion present in the polygluconate blocks maintained the integrity of the crosslinked beads investigated [20]. Gels crosslinked with barium cations have a higher Young's modulus than the calcium crosslinked gels attributed to a higher crosslinker density [21], further justifying the incorporation of barium as the crosslinking cation. 

 The main objectives of this study were to investigate the *in vitro *release of ciprofloxacin and diclofenac sodium as well as determine the antimicrobial activity of the PFD. It has been reported that the pH of a single periodontal pocket ranges from pH 2 to pH 9 [22]. This creates distinctly different chemical environments. Given that alginate displays pH responsive behaviour [23, 24], *in vitro* tests were conducted at both pH 4.0 and 6.8. The drug-loaded fibres were tested against *Escherichia coli* (*E. coli*), *Enterococcus faecalis* (*E. faecalis*), and *Streptococcus mutans* (*S. mutans*). *E. faecalis* and *E. coli* are examples of Gram-positive and Gram-negative facultative anaerobes, respectively, which have been found to be present in periodontitis. *S. Mutans* has been used on numerous occasions to test the antimicrobial activity of a device formulated for the treatment of PD [25–27].

## 2. Materials and Methods

### 2.1. Materials

Sodium alginate (Protanal LF 10/60) was purchased from FMC Biopolymer, Philadelphia, USA. Ciprofloxacin and diclofenac sodium were sourced from Sigma-Aldrich, Steinheim, Germany, and St. Louis, Missouri. Barium chloride 2-hydrate was procured from Saarchem, Pty (Ltd), Unilab, Wadeville, South Africa. Glycerol (*M*_*w*_ = 92.1 g/mol) was purchased from Rochelle Chemicals, Johannesburg, South Africa. Dialysis tubing cellulose membrane, average flat width of 33 mm, was acquired from Sigma-Aldrich, Steinheim, Germany. Tryptone Soya agar (TSA) was obtained from Oxoid, Hamphire and Hants, UK, respectively. Stock cultures of micro-organisms were obtained from Davies Diagnostics, South Africa. Buffering components, potassium dihydrogen orthophosphate, sodium hydroxide pellets, and hydrochloric acid, were correspondingly obtained from, Ace (Johannesburg), Merck (Wadeville), and Rochelle (Johannesburg). Double deionised water was obtained from a Milli-Q system (Milli-Q, Millipore, Johannesburg). UPLC grade acetonitrile and methanol were purchased from Romil Pure Chemistry, distributed by Microsep, Johannesburg. Formic acid (99%) was purchased from Merck, Wadeville. Sample vials (2 mL pre-silt, PTFE/silicone septa, screw top), and the Aquity UPLC Bridged Ethyl Hybrid (BEH) Shield RP_18_ column was procured from Waters, USA.

### 2.2. Formulation of Fibres

**Figure 1:**
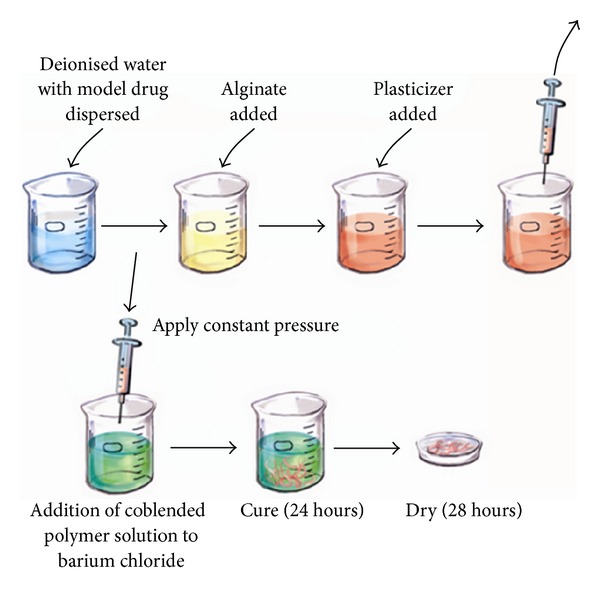
Schematic representation of preparation of the fibres.

The orodurable fibers were formulated using a simplified technique as reported elsewhere by the authors [27]. Briefly, the model drugs, ciprofloxacin (200 mg) and diclofenac sodium (500 mg), were dispersed in 25 mL deionised water. Sodium alginate was added to the drug solution prepared above to reach a final concentration of 3.14% w/v alginate followed by the addition of glycerol (22.54 mL) and was stirred overnight to achieve a homogenous mixture. The mixture was then loaded into a 10 mL syringe fitted with an 18 G needle. A constant pressure was applied to the syringe plunger to extrude thread-shaped fibres into a 10% w/v barium chloride cross-linker solution. The fibres were allowed to cure for 24 hours and were thereafter washed twice with deionised water and air dried at ambient conditions (25°C) until a constant weight was reached (approx. for 48 hours). For simplification, the process is illustrated step-by-step in Figure 1.

### 2.3. Characterisation of Morphological Transitions

Approximately 1 cm fibres were secured on carbon tape, attached to sample holders, and sputter coated (Spi-Module Sputter Coater, Apollo Scientific, Pennsylvania, USA) with gold for 90 seconds. Samples were then mounted in a scanning electron microscope (SEM) (Phenom Microscope, FEI Company, Hillsboro, Oregon, USA).

### 2.4. Characterisation of Vibrational Transitions

Chemical interactions between polymer, crosslinking salt, plasticiser, and model drugs were investigated using Fourier Transform Infrared (FT-IR) Spectroscopy using a FT-IR Spectrometer (PerkinElmer Inc., Waltham, Massachusetts, USA). The reference spectrum used was air. Samples tested included drug-free and drug-loaded polymeric fibres. Changes in vibrational absorption occur as new bonds are formed altering the infra red spectra which were qualitatively compared against one another. Formulation components and model drugs were run individually such that the sample spectra were compared to standard spectra.

### 2.5. Method Development for the Codetection of Ciprofloxacin and Diclofenac Sodium

Ciprofloxacin and diclofenac sodium absorb UV light within the same range, *λ*_max⁡_ = 278 nm and *λ*_max⁡_ = 276 nm; respectively, therefore, an ultraperformance liquid chromatography (UPLC) method to simultaneously detect the model drugs was developed using a Waters Aquity Performance LC system (Waters, Milford, MA, USA) coupled with a photodiode array detector (PDA). The UPLC was fitted with an Aquity UPLC Bridged Ethyl Hybrid (BEH) Shield RP_18_ column, with a pore size of 1.7 *μ*m. A gradient method with a run time of 3 minutes, depicted in Table 1, was developed using acetonitrile and 0.1% v/v formic acid in double deionised water as the mobile phases. Samples were filtered, using 0.22 *μ*m standard Isopore polycarbonate syringe filters, into 2 mL vials. The vials were placed in the UPLC sample holder. The injection volume was standardised at 2 *μ*L per injection.

### 2.6. Determination of Drug Entrapment of Optimised Fibres

Model drugs, ciprofloxacin, and diclofenac sodium, were dissolved in PBS pH 8 to generate standard calibration curves for subsequent determination of the drug entrapped in the optimised fibre. Serial injections (2 *μ*L) of varying concentrations of drug were injected, and the area under the curve (AUC) was calculated for peaks at 0.8 and 1.6 minutes for ciprofloxacin and diclofenac sodium, respectively. Approximately, 25 mg of fibres were submerged in 100 mL PBS pH 8 and stirred until the fibres had disintegrated. The samples were then filtered through a 0.22 *μ*m filter into a 2 mL sample vial. A 2 *μ*L sample was injected, and UV absorbance was detected at *λ* = 278 nm. The area under the curve was calculated and compared to the calibration curve to determine the concentration of model drug entrapped within the fibres.

### 2.7. Determination of Drug Release from PFD

Dissolution studies were conducted in 0.2 M phosphate buffer solution (PBS) at pH 4.0 and 6.8. PBS pH 4.0 and pH 6.8 were prepared according to the United States Pharmacopeia 23 (USP, 1995) recommendations [28]. Simulated pockets were designed using cellulose membrane dialysis tubes, 3.3 cm in diameter and 5 cm in length. Ciprofloxacin- and diclofenac-loaded fibres were weighed and placed within a simulated pocket. Either ends were secured with a nondegradable nylon based thread. The simulated pocket was placed in 20 mL PBS in a 50 mL glass jar and left in a shaking incubator (50 rpm) (Orbital Shaker Incubator, LM-530, Lasec Scientific Equipment, Johannesburg, South Africa) for 10 days. The drug release studies were conducted in triplicate. Samples were removed on days 0, 1, 2, 4, 6, 8, and 10 and frozen at −7°C until analysis. Samples were thawed at room temperature and filtered using a 0.22 *μ*m syringe filter into a 2 mL sample vial. Once all the samples were filtered and loaded, a sample set was run, and the autosampler injected 2 *μ*L of each sample according to the specified method. Each sample was tested in triplicate. Standard samples were run after the dissolution samples had been tested. For comparison, standard samples contained a known quantity of ciprofloxacin and diclofenac sodium dissolved in a 50 : 50 solution of doubled deionised water and acetonitrile. Serial dilutions with varying concentrations of drugs were injected. The areas under the curve for the relevant peaks were calculated and plotted against concentration of drug to construct a calibration curve, against which the dissolution samples were compared.

### 2.8. Agar Diffusion Studies

Agar diffusion studies were conducted as preliminary qualitative assays to screen the antimicrobial activity of the fibres prepared. Agar plates where 100 *μ*L of culture, and 20 mL of molten Tryptone Soya agar (TSA) were aseptically prepared. The plates inoculated with *S. mutans* were prepared with 2.5% v/v sheep's blood in Mueller Hilton agar. Samples of ciprofloxacin/diclofenac-loaded and drug-free fibres were transferred onto the surface of the solidified agar. The plates were placed at 4°C for 1 hour, allowing the drug to diffuse from the fibre into the agar, after which they were incubated at 37°C for 24 hours. Iodonitrotetrazolium chloride (INT) indicator dye (0.04 mg/mL) solution was sprayed onto inoculated plates to visualise the inhibition zones. INT in the presence of dehydrogenase changes colour to purple red. Dehydrogenase enzyme is present in metabolically active micro-organisms. The zone of inhibition was measured, from the fibre to the edge where microbial growth was visible, comparing fibres loaded with ciprofloxacin and diclofenac sodium to the control (drug free fibres). The zone of inhibition was asymmetrical surrounding the fibre; therefore, four readings were recorded, and the mean value was used for comparing the formulations. Agar diffusion studies were conducted in triplicate for each test microbe.

### 2.9. Minimum Inhibitory Concentration (MIC) Assays

The MIC microdilution bioassay was used to quantitatively assess the antimicrobial activity of the PFD. Aseptically, 100 *μ*L of distilled water was added to each well of a 96-well microtitre plate, followed by a 100 *μ*L of dissolution samples (diluted 1 : 10 with sterile water) which were added to the first row of the microtitre plates. Doubling serial dilutions were performed. Each dissolution sample was tested in duplicate. Bacterial cultures were subcultured from stock agar plates and grown in Tryptone Soya broth overnight to confirm viability. Cultures were diluted 1 : 100 in fresh Tryptone Soya broth, yielding an approximate inoculum size of  1 × 10^8^ colony forming units (CFU)/mL. Cultures (100 *μ*L) were added to all wells. Microtitre plates were sealed with an adhesive film to prevent the loss of any volatile samples and incubated for 24 hours at 37°C. Assays inoculated with *S. mutans* were placed in an anaerobic candle jar and incubated for 24 hours. After incubation, INT (40 *μ*L) was added to each well, and 6 hours later the MIC results were read as the lowest concentration of sample to inhibit microbial growth. Ciprofloxacin and diclofenac positive controls (0.1 mg/mL) were prepared by dissolving the drug in sterile water. Ciprofloxacin was tested to confirm the antimicrobial activity of the antibiotic used while diclofenac sodium was examined to confirm that this drug has no inherent antimicrobial activity. Equal quantities of the solutions were combined to test if the antimicrobial activity of ciprofloxacin was affected by diclofenac sodium. PBS pH 4.0 and 6.8 were analysed to ensure that the buffers used had no antimicrobial activity and that they were not contaminated. Broth with and without culture was assessed to validate, respectively, that the broth could support the growth of culture and that it was not contaminated.

### 2.10. Characterisation of Hydration Dynamics Behaviour

Once fibres were submerged in PBS, the matrices displayed diverse behaviour depending on the pH of the solution. To investigate the hydration behaviour of the optimised formulation, drug-free and drug-loaded fibres were separately assessed in 20 mL of PBS pH 4 or pH 6.8 and placed in a shaking incubator (Orbital Shaker Incubator, LM-530, Lasec Scientific Equipment, Johannesburg, South Africa) for 10 days. Samples were removed on days 0, 1, 2, 4, 6, 8, and 10. Once removed, the degree of swelling and erosion was determined through measuring the diameter of the fibre. The degree of hydration was calculated using the average diameter measured at three positions along the length of the fibre using digital calipers. The average diameter of the wet fibre was compared against the diameter of the dry fibre as a measure of the degree of hydration as represented in

(2) Degree of hydration =  Diameter of wet fibre−Diameter of dry fibreDiameter of dry fibre×100.

### 2.11. *In Vitro* Dissolution Studies

Dissolution apparatus used was as described in Section 3.5 for the analysis of drug release. Samples were removed on days 1, 2, 4, 6, 8, and 10 withdrawing 4 mL of solution which was retained at −4°C for further antimicrobial analysis. New samples were prepared for each time period tested. For comparative purposes, dissolution samples were collected from drug-free and ciprofloxacin/diclofenac-loaded fibres in PBS pH 4.0 and 6.8.

### 2.12. Static Lattice Atomistic Simulations

All modeling procedures and computations, including energy minimizations in molecular mechanics, were performed using HyperChem 8.0.8 Molecular Modeling System (Hypercube Inc., Gainesville, Florida, USA) and ChemBio3D Ultra 11.0 (CambridgeSoft Corporation,Cambridge, UK). The structure of alginate-Alginate_10_ (Alg_10_: ten oligosaccharide units) was generated (1,4-linked *β*-D-mannuronate: *α*-L-guluronate: 1 : 1) using the Sugar Builder Module on HyperChem 8.0.8. The structure of glycerol (Gly) was built with natural bond angles, and the models were initially energy-minimized using a MM+ force field, and the resulting structures were once again energy-minimized using the Amber 3 (Assisted Model Building and Energy Refinements) force field. The conformer having the lowest energy was used to create the polymer-plasticizer and polymer-crosslinker complexes. A complex of one molecule with another was assembled by disposing the molecules in parallel, and the same procedure of energy-minimization was repeated to generate the final models: Alg-Gly, Alg-Ba^2+^, and Alg-Gly-Ba^2+^. Full geometrical optimization was performed in vacuum employing the Polak-Ribiere conjugate gradient algorithm until an RMS gradient of 0.001 kcal/mol was reached. Force field options in the AMBER (with all hydrogen atoms explicitly included) and MM+ (extended to incorporate non-bonded restraints) methods were set as defaults [29].

## 3. Results and Discussion

### 3.1. Morphological and Surface Structure Analysis

**Figure 2:**
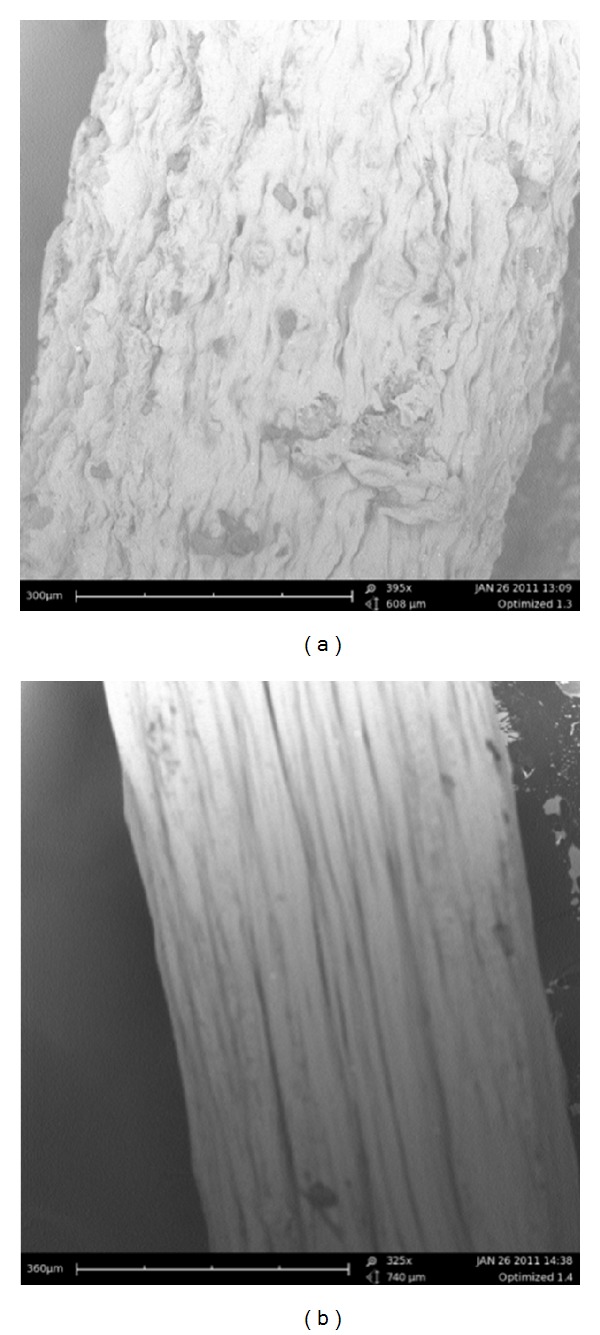
SEM images of (a) ciprofloxacin/diclofenac-loaded fibre and (b) drug-free fibres.

Analysis of SEM images revealed the interaction between the model drugs and the PFD as depicted in Figure 2. A uniform cylindrical filamentous structure is evident in the drug-free formulation (Figure 2(b)). Simultaneously, loading of ciprofloxacin and diclofenac sodium disrupts the fibre structure as seen in Figure 2(a). The surface of ciprofloxacin and diclofenac sodium-loaded fibre appears uneven and less aligned in structure compared to the drug-free formulation. 

### 3.2. Vibrational Transition Analysis

**Figure 3:**
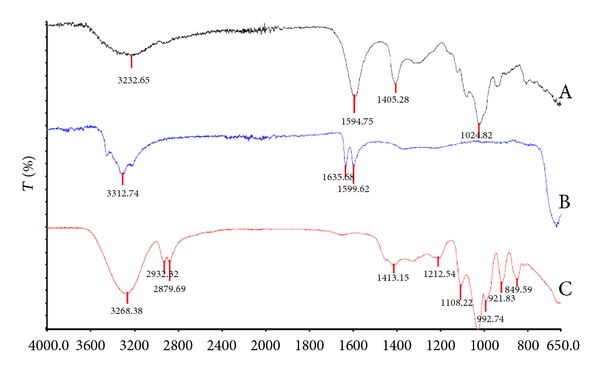
FT-IR spectrum of (A) alginate, (B) barium chloride 2-hydrate, and (C) glycerol.

**Figure 4:**
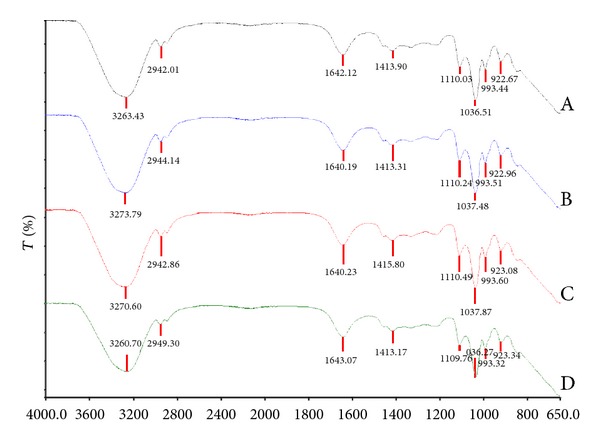
FT-IR spectrum representing (A) drug-free fibres, (B) ciprofloxacin-loaded fibres, (C) diclofenac sodium-loaded fibres, and (D) ciprofloxacin/diclofenac-loaded fibres.

Functional groups within molecules experience vibrational excitation and absorb infrared radiation corresponding to the vibrational energies. The absorption spectra are a blue print of the molecular structure and can therefore be used in the analysis of an unknown spectrum to reference spectrum [30]. The unknown spectra analysed were drug-free and drug-loaded co-blended crosslinked alginate based fibres. These spectra were compared against spectra of the individual components employed in the formulation of the fibres, namely, alginate, barium chloride 2-hydrate, and glycerol as depicted in Figures 3 and 4.

The analysis of the FTIR spectrum revealed broad peaks present in all fibres ranging from 3260.70 to 3273.79 cm^−1^, which characterises the presence of O-H groups. C-H stretching (2850–3000 cm^−1^) was present in the glycerol spectra with two peaks visible at 2932.32 cm^−1^ and 2879.69 cm^−1^. Similar peaks were present in all fibre formulations shifting towards slightly higher wavelengths. Two unknown peaks at 1635.68 cm^−1^ and 1599.62 cm^−1^ were present in the barium chloride spectra. The crosslinking between alginate and barium chloride was observed by Lawrie and coworkers, identifying the antisymmetric carbonyl vibration (1596 cm^−1^) has been most sensitive to the addition of barium chloride ions [31]. Similarly, in Figure 3, the peak present in the sodium alginate spectra at 1594.28 cm^−1^ was absent or had shifted in the spectra in Figure 4. A comparable peak at approximately 1640 cm^−1^ was observed in all four fibre formulations which may reflect the unknown peak from the barium chloride spectra or a shift in the carbonyl vibration present in sodium alginate. Furthermore, a shift in the bands to higher or lower energies may have occurred due to sodium alginate crosslinking with divalent ions which is dependent on the M/G monomer ratio [31]. Shifts in peaks at 1405.28 cm^−1^ and 1024.82 cm^−1^ in the sodium alginate spectrum to 1413.17–1415.80 cm^−1^ and 1036.27–1037.87 cm^−1^, respectively, further reflected in the interaction between barium chloride and alginate. The shift in peak at 1030 cm^−1^ was noted by Sakugawa and co-workers, reflecting a change in M monomer concentration in the presence of calcium ions, confirming the shifts in peaks evident between 1024.82 cm^−1^ and approximately 1037 cm^−1^ as an interaction between the polymer and crosslinking salt [32]. Incorporation of ciprofloxacin and diclofenac, individually or in combination within the fibre, did not result in any significant changes in the spectra when compared to the drug-free fibres. If no chemical interactions between the polymer and drug are evident, the release will depend on the hydrophobicity of the drug diffusing through the gel pores [17].

### 3.3. UPLC Method Development for the Codetection of Ciprofloxacin and Diclofenac Sodium

**Figure 5:**
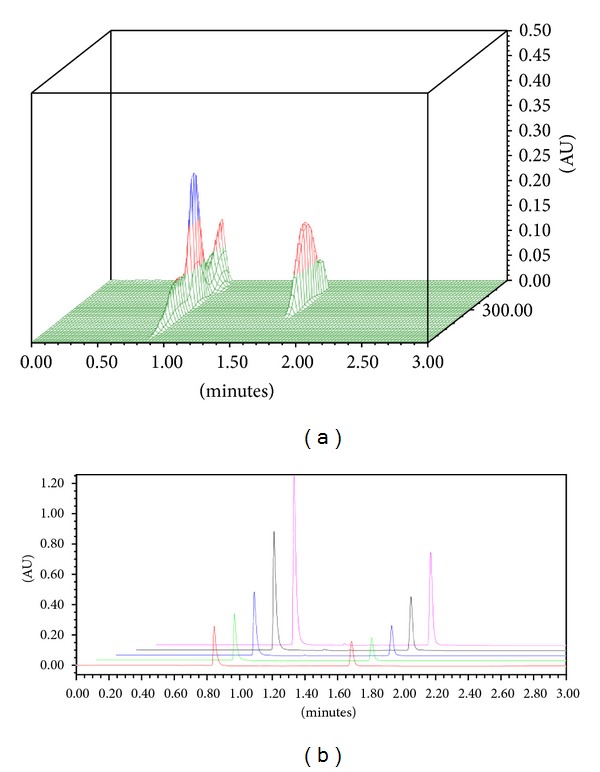
(a) PDA spectrum for the detection of ciprofloxacin and diclofenac sodium at approximately 0.8 and 1.7 minutes, respectively, and (b) overlaid chromatograms representing the serial dilutions where the area under the curve at 0.8 minutes and 1.7 minutes was used in determining the calibration curves for ciprofloxacin and diclofenac sodium, respectively.

The UPLC builds on the technology of high performance liquid chromatography (HPLC) analysing compounds with increased speed, sensitivity, and resolution while drastically reducing run times and has significantly improved processes within drug delivery [33]. Inclusion of ciprofloxacin and diclofenac within the same fibre necessitated the development of a chromatographic method for the codetection of these two drugs from dissolution samples. The reverse phase chromatographic method analyses the chemical structures affinity for the nonpolar stationary phase or the more polar mobile phase. Using a gradient method, the composition of the mobile phase was adjusted to ensure that both drugs produced narrow peaks without visible tailing and that these peaks were easily resolved from one another. The three-dimensional analysis of the PDA spectrum from *λ* = 220 – 400 nm identified that ciprofloxacin was eluted before diclofenac sodium with retention times of 0.8 minutes and 1.7 minutes, respectively, depicted in Figure 5(a). Adequate separation of the two peaks as well as the maximum peak for both drugs was between 270–280 nm; therefore, the channel was set at *λ* = 278 nm for analysis.

### 3.4. Drug Entrapment Efficiency of Fibres

The USP 1995 states that both peak height and area can be related to analyte concentration; however, peak areas have been considered a more accurate method for quantification. Resolution between peaks was sufficient; consequently, the peak areas were used to quantify the concentration of ciprofloxacin and diclofenac sodium in both standards and samples. The calibration curves for ciprofloxacin and diclofenac sodium were used to determine the drug entrapment within the optimised fibre, plotting the area under the curve (AUC) versus the concentration of either ciprofloxacin or diclofenac sodium. The incremental increases in absorbance and thus area are noted in Figure 5(b), representing an increase in concentration of drug with each standard sample injected. 

It was uncertain if both drugs would remain entrapped within the fibre when combined simultaneously in the same formulation. Drug entrapment samples that were taken from drug-loaded fibres dissolved in PBS pH 8 confirmed that both ciprofloxacin and diclofenac were successfully retained within the formulation at 79.43% (±3.68) and 95.78% (±1.93), respectively.

### 3.5. Swelling and Erosion Analysis

**Figure 6:**
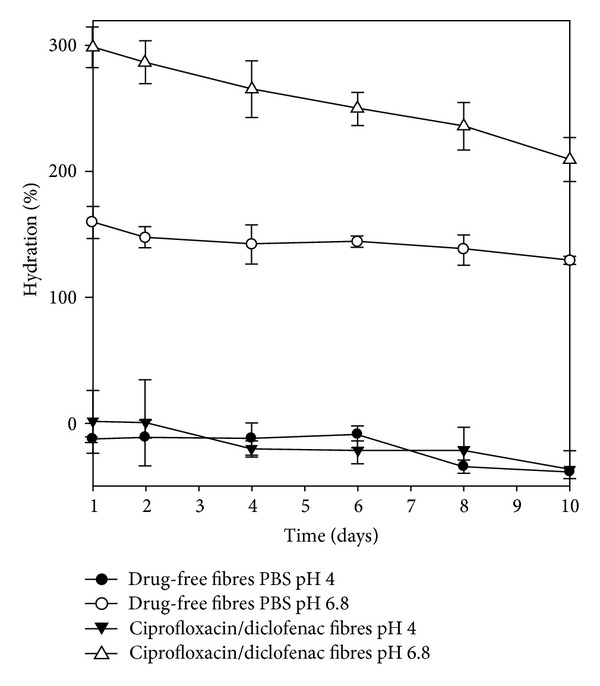
The hydration behaviour of drug-free and ciprofloxacin/diclofenac-loaded PFD over 10 days in PBS pH4 and 6.8 (*N* = 3).

Alginate interacts with divalent cations resulting in gelation or precipitation while a soluble salt complex is formed when monovalent cations interact with the polymer. Alginate based polymeric fibres initially swelled in PBS pH 6.8 up to 300% in the first day, after which the matrix eroded steadily over the next few days. In comparison, the fibre does not swell in PBS pH 4 but slowly erodes. Swelling of crosslinked alginate matrices in PBS is explained as a result of the divalent crosslinked ions being replaced by sodium ions present in solution, resulting in repulsion of the –COO– groups, chain relaxation, and swelling [20]. The degree of hydration varied considerably in PBS at pH 6.8 between the drug loaded and drug-free samples as depicted in Figure 6. Ciprofloxacin/diclofenac-loaded fibres swelled to almost twice as much as drug-free fibres. This may perhaps be ascribed to the degree of crosslinking and porosity of the matrix interacting with the sodium ions present in solution.

### 3.6. *In Vitro* Drug Release Behaviour

For accurate analysis, the analyte needs to be soluble in the mobile phases, hence for analysis of dissolution samples, standard samples of ciprofloxacin and diclofenac sodium were dissolved in water and acetonitrile at a ratio of 1 : 1, which was completely miscible with the mobile phases over the three minutes run time. The AUC was calculated and plotted against drug concentration giving calibration curves for ciprofloxacin and diclofenac sodium. In an attempt to create a pocket-like environment, fibres were placed in the dialysing tube as described in a study conducted by Mundargi and co-workers [34]. The dialysing tube was tied at either ends with nylon thread to prevent the escape of the fibre and then submerged in PBS. The dialysing tube used freely allows the passage of compounds with a molecular weight less than 12,000 g/mol. Drug interactions between ciprofloxacin and diclofenac affecting release kinetics as well as capacity of the fibre to retain these two drugs in sufficient concentrations were two major concerns of simultaneous drug entrapment. 

**Figure 7:**
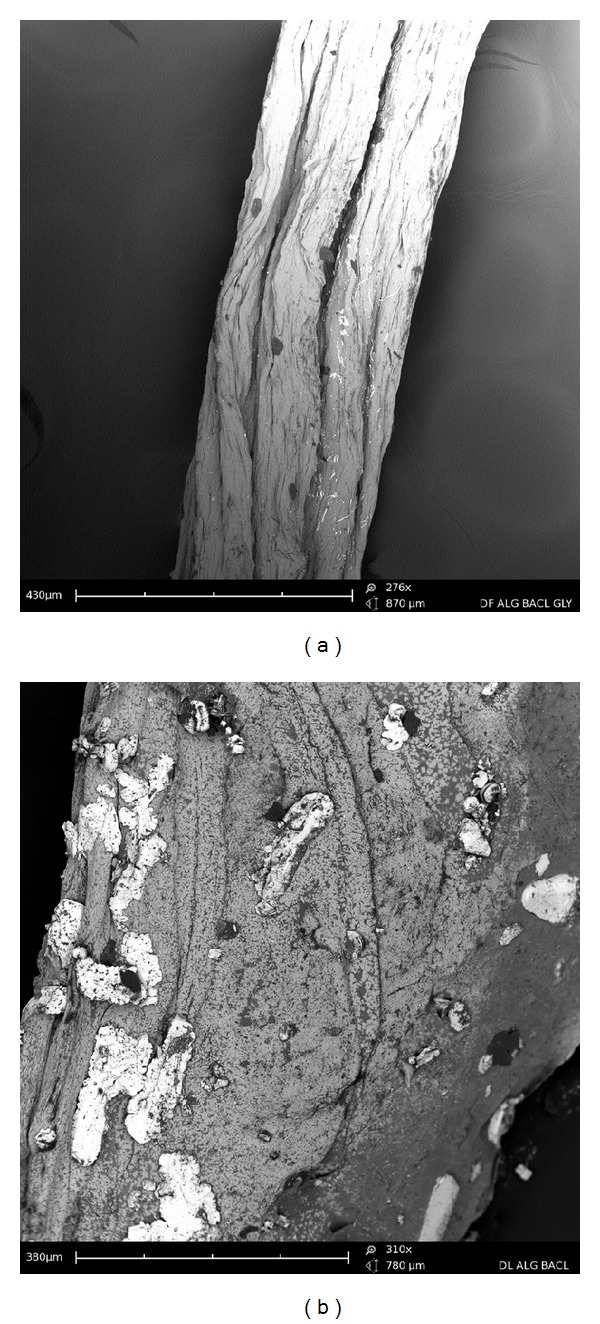
Drug-free fibres following 10-day dissolution study in PBS (a) pH 4.0 and (b) pH 6.8.

**Figure 8:**
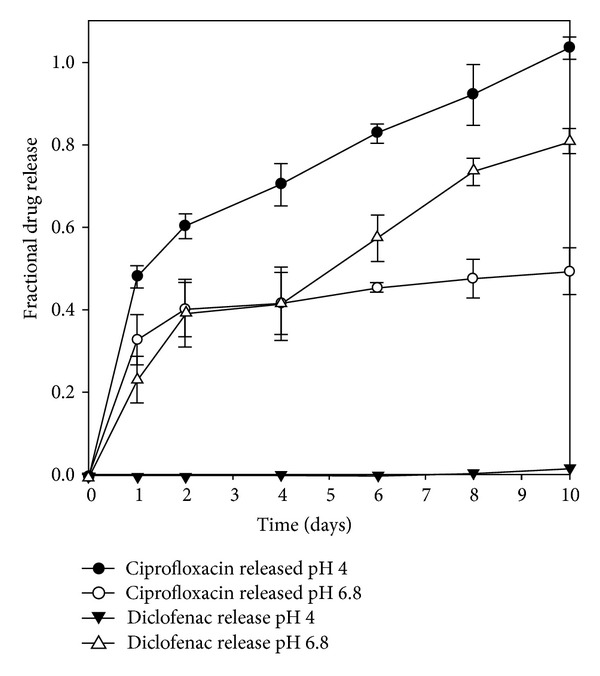
Simultaneous fractional drug release of ciprofloxacin and diclofenac sodium from the optimised fibre formulation (*N* = 3).

Drug release kinetics has been reported to depend on drug solubility, pH of dissolution medium, and changes in the crystalline structure of the matrix upon hydration [35]. Changes in the morphological structure of the fibres were assessed in SEM images of fibres following exposure to PBS 4 and 6.8 over 10 days (Figure 7). Examination of the fibres in PBS pH 4.0 showed that the shape of the fibre remains intact following dissolution (Figure 7(a)). Therefore, drug release from the PFD occurs as drug diffuses fibre in to the surrounding medium. Relaxation and swelling of the polymeric matrix are evident in Figure 7(b) representing samples exposed to PBS pH 6.8. Furthermore, the flakes or pieces attached to the surface of the fibres are indicative of surface erosion. This analysis confirms that drug release in PBS pH 6.8 occurs as a result of swelling and erosion leading to disruption of the polymeric matrix. Tønnesen and Karlsen describe the release of drug from calcium chloride crosslinked alginate as a “swelling-dissolution-erosion process” thereby modulating drug release [36]. However, a crosslinked alginate forms an insoluble matrix in an acidic medium releasing the drug via diffusion mechanisms. The fractional drug release over 10 days (Figure 8) confirmed that ciprofloxacin was consistently released over 10 days at both pHs tested. Ciprofloxacin, a weak basic drug, was released to a greater extent in PBS pH 4.0 as it is more soluble in acidic media compared to PBS pH 6.8 where it is less soluble. However, it is suggested that the swelling and relaxation of the fibre accounted for the ciprofloxacin's release in PBS pH 6.8. In contrast, diclofenac sodium, a weak acidic drug, released 80% of the entrapped drug in PBS pH 6.8. Exceptionally, low levels of diclofenac were released in PBS pH 4.0, which can be hypothesised as being due to the drugs poor solubility in acidic media in conjunction with reduced swelling and relaxation of the PFD in PBS pH 4.0. 

**Figure 9:**
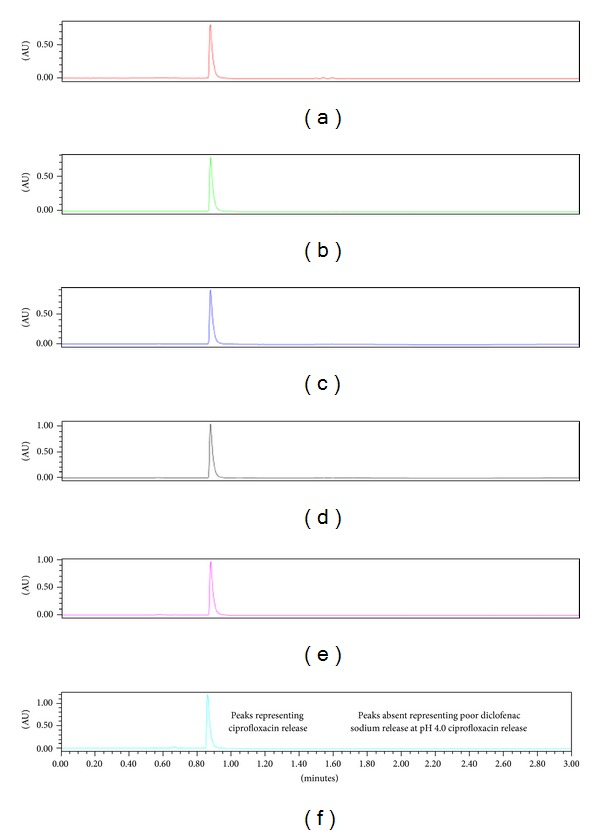
Stacked chromatograms of dissolution samples in PBS pH 4.0 on days (a) 1, (b) 2, (c) 4, (d) 6, (e) 8, and (f) 10 at *λ* = 278 nm. Noticeably, consequential increase in AU implies increase in drug release.

**Figure 10:**
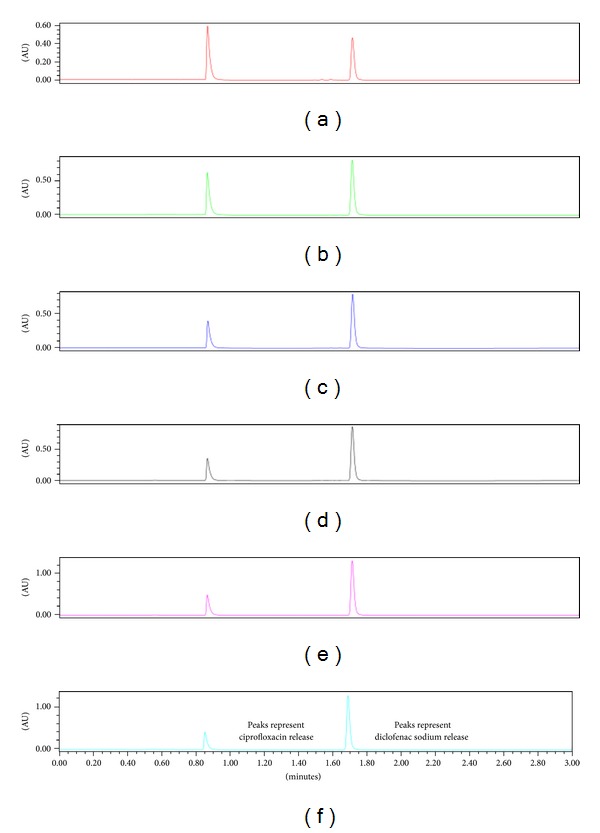
Stacked chromatograms for dissolution samples in PBS pH 6.8 on days (a) 1, (b) 2, (c) 4, (d) 6, (e) 8, and (f) 10 at *λ* = 278 nm. Noticeably, consequential increase in AU implies increase in drug release.

Diclofenac sodium was poorly released in PBS pH 4.0, but was steadily released in PBS pH 6.8 over the same time period. These results are visually represented in the stacked chromatograms representing drug release at each time point in PBS pH 4.0 and pH 6.8 correspondingly in Figures 9 and 10. The peak at approximately 1.7 minutes, characterising diclofenac release, is absent in Figure 9; however, it is present in the chromatograms at each time interval from day 1 to day 10 in Figure 10. Comparatively, peaks at approximately 0.8 minutes represent ciprofloxacin released at pH 4.0 and pH 6.8 and is present at each time interval tested. 

 The MDT for ciprofloxacin and diclofenac sodium in PBS pH 4.0 and 6.8 is summarised in Table 2. In comparison to the MDT for optimised fibres with either ciprofloxacin or diclofenac sodium loaded within the fibre, a notable reduction in MDT_10 days_ for both drugs at both pHs was noted. A reduction in MDT validated the proposition that the matrix retards the release of both drugs. 

### 3.7. Qualitative Studies Assessed Using Agar Diffusion Assays

Agar diffusion assays were used to investigate if the fibres contained sufficient ciprofloxacin and if the entrapped drug was released from the polymeric matrix to inhibit microbial growth. Ciprofloxacin/diclofenac-loaded fibres significantly inhibited the growth of all three test organisms while the control fibres having no drug showed no inhibitory activity. The zones of inhibition mean measurements are summarized in Table 3. The irregular zone of inhibition surrounding the fibre as well as the necessity to quantitatively measure the effectiveness of the fibre in releasing ciprofloxacin limits the use of this assay to qualitative purposes only.

### 3.8. Quantitative Studies Assessed Using MIC Assays

The purpose of performing MIC analysis on the PFD was to determine if sufficient ciprofloxacin was released from the fibre at each time point. Furthermore, it was necessary to quantify the antimicrobial activity of the prolonged delivery device against the three test pathogens that were screened for activity in the diffusion assays. The relatively constant MIC over the 10-day sampling period for dissolution studies confirms that sufficient ciprofloxacin was released from ciprofloxacin and diclofenac-loaded fibres to inhibit the growth of all three micro-organisms at both pH 4.0 and 6.8 (Table 4). The MIC ranges at pH 4.0 for *E. coli*, *E. faecalis, *and* S. mutans* were 0.5–0.8 *μ*g/mL, 0.4–1.1 *μ*g/mL, and 0.7–2.1 *μ*g/mL, respectively. At pH 6.8, similar efficacies (0.3–0.5 *μ*g/mL for *E. coli *and* E. faecalis* and 0.6–1.0 *μ*g/mL for *S. mutans*) were observed. Very little variation in efficacy was evident over the 10-day sampling period attesting to the steady concentrations of ciprofloxacin being released. Furthermore, little variation in efficacy was evident between the different pHs tested suggesting that that the antimicrobial effects noted are somewhat independent of the pH of the PBS. The PBS controls at pH 4.0 and 6.8 as well as drug-free fibres demonstrated no inhibition of growth, confirming that the antimicrobial activity noted with the drug-loaded fibre is not attributed to the buffer or formulation components correspondingly. The broth with and without culture confirmed the test organism viability and broth sterility, respectively. In comparison, the positive control for ciprofloxacin (MIC value range 2.5–10.0 *μ*g/mL) demonstrated susceptibility of the test organism. Diclofenac conferred no antimicrobial activity (Table 4). 

### 3.9. Molecular Mechanics Assisted Model Building and Energy Refinements

Molecular mechanics energy relationship (MMER), a method for analyticomathematical representation of potential energy surfaces, was used to provide information about the contributions of valence terms, noncovalent Coulombic terms, and noncovalent van der Waals interactions in drug incorporation and release effects. The MMER model for potential energy factor in various molecular complexes can be written as

(3) Emolecule/complex  =VΣ=Vb+Vθ+Vφ+Vij+Vhb+Vel,

where *V*_Σ_ is related to total steric energy for an optimized structure, *V*_*b*_ corresponds to bond stretching contributions (reference values were assigned to all of a structure's bond lengths), *V*_*θ*_ denotes bond angle contributions (reference values were assigned to all of a structure's bond angles), *V*_*φ*_ represents torsional contribution arising from deviations from optimum dihedral angles, *V*_*ij*_ incorporates van der Waals interactions due to nonbonded interatomic distances, *V*_hb_ symbolizes hydrogen-bond energy function, and *V*_el_ stands for electrostatic energy [37].

#### 3.9.1. Formulation and Crosslinking of Plasticized-Alginate Fibers

**Figure 11:**
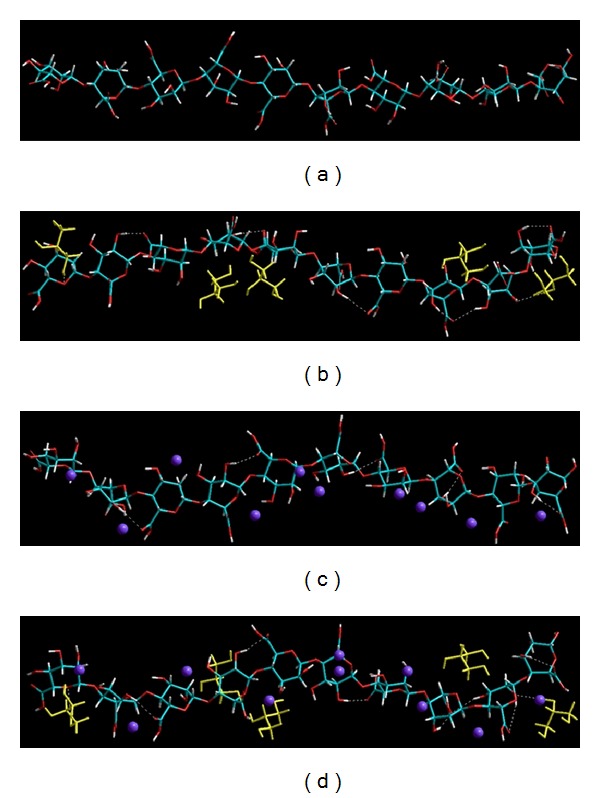
Visualization of energy minimized geometrical preferences of (a) alginate (10 monosaccharide units); (b) alginate-glycerol; (c) Alginate-Ba^2+^; and (d) Alginate-Glycerol-Ba^2+^ showcasing the intra- and intermolecular interactions in crosslinked-plasticized-alginate fibers after molecular simulations in vacuum. Glycerol molecules are rendered as tubes in yellow, and barium ions are rendered spherically in violet. Color codes for elements: C (cyan), O (red), N (blue), and H (white).

The present work riveted around the fabrication of a polysaccharide (alginate) based macrofibrous system integrating a unique combination of plasticizer (glycerol) and crosslinker (divalent barium ions). The energy-minimized geometrical conformations are depicted in Figure 11, and the corresponding energy equations are represented by (4)–(8). In accordance with the experimental sequence, alginate solution was first plasticized by the addition of glycerol and thereafter crosslinked in barium chloride solution in form of fibers. Although the drug release analysis was conducted on crosslinked-plasticized fiber, we hereby present an account of the effect of incorporation of the individual component on the fibers' performance.

Consider

(4) EALG-10=  74.426VΣ=5.204Vb+32.485Vθ+34.168Vφ+30.709Vij−28.142Vel,

(5) EGLY-5=14.315VΣ=0.230Vb+1.345Vθ+  10.615Vφ+2.185Vij−0.065Vhb,

(6) EALG-GLY5=22.558VΣ+5.698Vb+  32.688Vθ+47.908Vφ  +17.697Vij−1.454Vhb−79.979Vel,

(7) EALG-Ba102+=  38.839VΣ=  5.662Vb+  31.068Vθ+  40.489Vφ+36.363Vij−74.744Vel,

(8) EALG-GLY5-Ba102+  =14.917VΣ  =5.641Vb+27.792Vθ  +38.07Vφ+24.932Vij  −1.456Vhb−80.065Vel.

The incorporation of glycerol to alginate conceded the standards of polymer-plasticizer combination, both kinetically and thermodynamically. Kinetically, glycerol dissolved rapidly into the alginate solution, and in the thermodynamic sense, the free energy of mixing or the change on total steric energy of complex was negative (−66.183 kcal/mol; (4)–(6); Table 5) thereby forming a highly stable preferential molecular composite with intra- and intermolecular H-bonds ranging from 2.2052 A° to 3.0464 A° (avg. 2.4647 A°). Furthermore, glycerol displayed strong interactions with the polysaccharide as displayed in Figure 11. Alginates are known to exhibit interactions with various divalent metal ions, such as Ca^2+^ and Ba^2+^, where they display a unique crosslinking and complexation phenomenon in the form of in intramolecular as well as intermolecular interaction depending on the concentration of the cations [38]. It is evident from Table 5 that Alg-Ba^2+^ was energetically stabilized by 35.587 kcal/mol ((4) and (7)) as compared to algorithm because of strong electrostatic interactions (Δ*E* = −46.602 kcal/mol) along with high torsional energy (Δ*E* = 6.321 kcal/mol) resulting in the establishment of a strained complex arrangement—with reduced intramolecular H-bond lengths ranging from 2.208 A° to 3.3595 A° (avg. 2.2996 A°)—due to calcium crosslinking thereby limiting the complete interaction as observed with the noncrosslinked structure (Figure 11). These results conform to the previously reported research work by Braccini and Peŕez, wherein the polyguluronates displayed enhanced strength and stereospecificity in close proximity with Ca^2+^ through “Egg-box model” [39].

The crosslinked-plasticized polymeric matrix, Alg-Gly-Ba^2+^, formed by ionic crosslinking and subsequent gelation of already plasticized alginate displayed a well-organized structure with formation of several intra- and inter-molecular H-bonds (although at different positions as compared to bimolecular structures) having bond length ranging from 2.2051 A° to 3.1906 A° with (avg. 2.5486 A°). The trimolecular complex is stabilized by 38.237 kcal/mol which is in-between the stabilization energies of the corresponding bimolecular structures, Alg-Gly and Alg-Ba^2+^, demonstrating the conformational stability and compatibility of the three molecules in conjugation with each other. Interestingly and unexpectedly, both bonding and nonbonding energy values were stabilized with negative Δ*E* values endorsing the thermodynamic and steric rationality of the selected plasticizer and crosslinker combination for alginate fibers.

#### 3.9.2. Drug Release from Crosslinked-Plasticized-Alginate Fibers

In this work, we performed molecular mechanics simulations of polymer-plasticizer-crosslinker composite wherein we calculated the energy changes involved in the amalgamation of alginate, glycerol, and barium cations by derived energy relationships based on molecular interactions and thus predicted the influence of the independent variables on the drug release profile of the formed polymer structure. Additionally, based on the various intra- and inter-molecular interaction energy terms such as van der Waals forces (vdWf), we provide an assumption that the drug release may further be dependent on (1) the matrix rigidity and (2) the matrix flexibility inherent to the polymer fragments. The complexation of 10-monosaccharide units' alginate chain with five glycerol molecules leads to a decrease in vdWf by ~15 kcal/mol (Table 5) which may result in reduction of the rigidity of the matrix and increasing the flexibility of the polymer chains. Consequently, an increase in glycerol-mediated plasticization may minimize the insoluble component of the crosslinked polymer.

With respect to the extrapolation of the influence of crosslinking on the drug release, although the total energy was stabilized by ~36 kcal/mol in Alg-Ba^2+^, we observed an increase of vdW energy by 5.654 kcal/mol (Table 5) which may be responsible for an increase in the rigidity due to an increase in cohesivity with increase in barium ion concentration. Apart from dispersion forces, Alg-Ba_10_^2+^ was not stabilized by H-bonding, and even the electrostatic forces were less stabilized, as compared to Alg-Gly_5_. The simulation profile demonstrated that the cation-induced crosslinks may lead to the creation of disturbance at high crosslinking levels causing a distortion of bond angles and torsional fragments from their symmetry values. This eventually can originate the formation of a strained and rigid-framework (Figure 11(b)). Furthermore, the accumulation of cohesion forces among the side-by-side polymer fragments chains, due to intermolecular crosslinking, may induce an axial stress. This rigidification of the crosslinked matrix can provide a realistic explanation for the experimentally observed decrease in drug release with increase in crosslinking.

The prospective effect of variation of levels of plasticizer and crosslinker on the release properties of alginate fibers can be explained on similar lines. The matrix hardness in case of Alg-Gly5-Ba_10_^2+^ seems to be tilted more in favor of Alg-Gly_5_ with ΔVdw of Alg-Gly5-Ba_10_^2+^ (~13 kcal/mol) close to that of Alg-Gly_5_. An increase in crosslinking may significantly decrease the dissolution medium's transport through the matrix and may even push out the already present glycerol, thereby hindering the impact of plasticizer. In the opposite way, an increase in plasticizer content may result in weakening of Ba^2+^ induced intermolecular interactions increasing the available “free volume” causing an increase in their flexibility by allowing the polymer molecules to move more freely [40]. Nevertheless, the effect of addition of Ba^2+^ on Alg-Gly_5_ is evident from the fact that all the energy values attained stabilization with even stretching and torsional contributions playing their part in structural optimization. This confirms our effort that an optimized alginate fiber formulation having desired release properties can be attained by varying the concentrations of plasticizer and crosslinking agent.

## 4. Conclusions

Crosslinked alginate matrices demonstrate pH-responsive behaviour; therefore, the polymeric fibre device was tested at pH 4.0 and 6.8. Drug release at pH 4.0 occurred as a result of drug diffusing through the polymeric fibres. However, at pH 6.8 the disruption of the fibre structure led to drug release as a consequence of the swelling and erosion of the matrix. Ciprofloxacin was sufficiently released from the drug-loaded fibres inhibiting growth of *E. coli*, *E. faecalis,* and *S. mutans* over 10 days. Dissolution samples where drug-free fibres were tested proved that the formulation without ciprofloxacin did not demonstrate activity against microbes. The results thus far are promising, therefore advocating further preclinical and clinical analyses of the fibres.

## Figures and Tables

**Table 1:** Gradient method used in the separation of ciprofloxacin and diclofenac sodium indicating mobile phase concentrations and flow rate at various time periods over the 3 minute run time.

Time (min)	Flow rate (mL/min)	0.1% v/v Formic acid solution	Acetonitrile
Initial	0.5	85%	15%
1.00	0.5	20%	80%
3.00	0.5	85%	15%

**Table 2:** MDT_10  Days_ for ciprofloxacin and diclofenac release in PBS pH 4.0 and pH 6.8.

Days	MDT_10 days ciprofloxacin_		MDT_10 days diclofenac sodium_	
	pH 4.0	pH 6.8	pH 4.0	pH 6.8
0	0	0	0	0
1	0.24 ± 0.01	0.21 ± 0.01	0	0.11 ± 0.01
2	0.91 ± 0.08	0.74 ± 0.03	0	0.62 ± 0.01
4	2.11 ± 0.17	1.21 ± 0.04	0.012 ± 0.00	1.18 ± 0.23
6	4.14 ± 0.22	1.64 ± 0.06	0.002 ± 0.00	2.87 ± 0.14
8	6.45 ± 0.79	3.18 ± 0.19	0.04 ± 0.00	5.15 ± 0.62
10	9.31 ± 0.62	4.29 ± 0.88	0.15 ± 0.01	7.28 ± 0.98

**Table 3:** Mean zone of inhibition measurements for fibres against three test organisms.

Test organism	Zone of inhibition (mm)	
	Drug loaded	Control (drug free fibre)
*E. coli* (ACC 8739)	14.96 ± 0.94	0.00
*E. faecalis* (ATCC 29212)	14.83 ± 0.32	0.00
*S. mutans* (NCTC 10919)	17.79 ± 2.65	0.00

**Table 4:** MIC results for ciprofloxacin/diclofenac-loaded fibres from dissolution samples in PBS pH 4.0 and 6.8 over 10 days.

Dissolution samples	PBS pH 4.0			PBS pH 6.8		
	*E. coli *	*E. faecalis *	*S. mutans *	*E. coli *	*E. faecalis *	*S. mutans *
Day 1	0.5	0.4	0.7	0.4	0.4	0.8
Day 2	0.6	0.6	1.2	0.5	0.5	1.0
Day 4	0.7	1.1	1.4	0.4	0.4	0.8
Day 6	0.8	0.8	1.7	0.3	0.3	0.6
Day 8	0.5	0.4	1.9	0.5	0.5	0.9
Day 10	0.5	0.5	2.1	0.5	0.5	1.0

Controls						
Ciprofloxacin	2.5	2.5	10.0	2.5	2.5	10.0
Diclofenac	NI	NI	NI	NI	NI	NI
Drug free fibres (Day 1–10)	NI	NI	NI	NI	NI	NI
PBS pH 4.0	NI	NI	NI	NI	NI	NI
Broth with culture	NI	NI	NI	NI	NI	NI
Broth	NG	NG	NG	NG	NG	NG

NI: no inhibition; NG: no growth. All values are expressed as *μ*g/mL.

**Table 5:** Calculated energy parameters (kcal/mol) of polymer-plasticizer, polymer-crosslinker, and crosslinked-plasticized polymer assemblies.

Name	ΔEnergies^a^						
	ΔTotal^b^	ΔBond^c^	ΔAngle^d^	ΔDihedral^e^	ΔVdw^f^	ΔH-bond^g^	ΔElectro^h^
Alg-Gly_5_^i^	−66.183	0.264	−1.142	3.125	−15.197	−1.389	−51.837
Alg-Ba_10_^2+^ ^j^	−35.587	0.458	−1.417	6.321	5.654	0.000	−46.602
Alg-Gly_5_-Ba_10_^2+^ ^k^	−38.237	−0.251	−4.621	−13.032	−13.616	−1.391	−5.321

^a^Δ*E*_binding_ = *E*(Host·Guest) – *E*(Host) – *E*(Guest); ^b^total steric energy for an optimized structure; ^c^bond stretching contributions; ^d^bond angle contributions; ^e^torsional contribution; ^f^van der Waals interactions; ^g^hydrogen-bond energy function; ^h^electrostatic energy; ^i^five glycerol molecules in conjugation with an alginate chain; ^j^ten barium ions in conjugation with an alginate chain; ^k^five glycerol molecules and ten barium ions in conjugation with an alginate chain.
